# Barriers and facilitators to improve safety and efficiency of the ICU discharge process: a mixed methods study

**DOI:** 10.1186/s12913-017-2139-x

**Published:** 2017-04-04

**Authors:** Nelleke van Sluisveld, Anke Oerlemans, Gert Westert, Johannes Gerardus van der Hoeven, Hub Wollersheim, Marieke Zegers

**Affiliations:** 1 Radboud University Medical Center, Radboud Institute for Health Sciences, IQ Healthcare, P.O. Box 9101, 6500 HB Nijmegen, The Netherlands; 2 Department of Intensive Care Medicine, Radboud University Medical Center, P.O. Box 9101, 6500 HB Nijmegen, The Netherlands

**Keywords:** Intensive care, Critical care, Intensive care unit, Patient readmission, Mortality, Discharge practices

## Abstract

**Background:**

Evidence indicates that suboptimal clinical handover from the intensive care unit (ICU) to general wards leads to unnecessary ICU readmissions and increased mortality. We aimed to gain insight into barriers and facilitators to implement and use ICU discharge practices.

**Methods:**

A mixed methods approach was conducted, using 1) 23 individual and four focus group interviews, with post-ICU patients, ICU managers, and nurses and physicians working in the ICU or general ward of ten Dutch hospitals, and 2) a questionnaire survey, which contained 27 statements derived from the interviews, and was completed by 166 ICU physicians (21.8%) from 64 Dutch hospitals (71.1% of the total of 90 Dutch hospitals).

**Results:**

The interviews resulted in 66 barriers and facilitators related to: the intervention (e.g., feasibility); the professional (e.g., attitude towards checklists); social factors (e.g., presence or absence of a culture of feedback); and the organisation (e.g., financial resources). A facilitator considered important by ICU physicians was a checklist to structure discharge communication (92.2%). Barriers deemed important were lack of a culture of feedback (55.4%), an absence of discharge criteria (23.5%), and an overestimation of the capabilities of general wards to care for complex patients by ICU physicians (74.7%).

**Conclusions:**

Based on the barriers and facilitators found in this study, improving handover communication, formulating specific discharge criteria, stimulating a culture of feedback, and preventing overestimation of the general ward are important to effectively improve the ICU discharge process.

**Electronic supplementary material:**

The online version of this article (doi:10.1186/s12913-017-2139-x) contains supplementary material, which is available to authorized users.

## Background

Discharging patients from an intensive care unit (ICU) to a general ward is a high-risk event due to the number, complexity and acuity of the patients’ medical conditions and the significant reduction in monitoring [1]. Suboptimal clinical handover may result in poor continuity of care and in adverse patient outcomes leading to ICU readmissions and patients’ death [2–4]. In the Netherlands, the percentage of ICU readmissions is 7.5% [5], compared to 2.4 to 6.3% in the United States [4, 6] and 7.0% in Canada [7]. Of these ICU readmissions, percentages ranging from 11.8 to 21.8% are potentially preventable [8, 9]. In the Netherlands, reported percentages of in-hospital mortality of ICU patients range from 6.7 to 17.3% [5, 10], compared to 4.1% in the United States [6] and 9.4% in Canada [7].

A well-organised ICU discharge process includes the discharge decision, planning and preparation for discharge, safe transport of the patient, and structural follow-up or care after ICU discharge. The organisation of the ICU discharge process differs among hospitals (van Sluisveld N, Bakhshi-Raiez F, de Keizer NF, Holman R, Westert GP, Wollersheim H, van der Hoeven JG, Zegers M. Variation in rates of ICU readmissions and post-ICU in-hospital mortality and their association with ICU discharge practices, unpublished) [11]. Moreover, rates of ICU readmissions and post-ICU in-hospital mortality vary between hospitals, indicating room for improving the ICU discharge process.

Factors influencing an effective ICU discharge process are well known [12], and the number of interventions aiming to improve the handover of ICU patients, such as the use of handover forms and liaison nurses, is growing [13]. While the process seems straightforward, implementing quality improvement interventions is very difficult [14]. Systematic understanding of the factors that influence implementation of ICU discharge improvement practices is lacking. Insight is necessary to optimise the use of these practices in daily practice and ultimately to improve patient outcomes.

The purpose of this study was to gain insight into barriers to and facilitators for the implementation and use of ICU discharge practices.

## Methods

### Study design and setting

A mixed methods design was adopted, including qualitative methods, individual and focus group interviews, and quantitative methods, an online questionnaire survey sent to all Dutch ICU physicians (*n* = 761) working in all Dutch hospitals (*n* = 90). The questionnaire was used to quantify the results of the interviews. We used the COREQ guideline to design and report the qualitative research (Additional file 1) [15].

Hospitals in the Netherlands can be categorised into three types: general, teaching and academic hospitals. The ICUs in the Netherlands are organised in a closed format system with intensivists who coordinate care together with the admitting specialist. Three levels of care are defined, based on annual patient volume, number of ICU beds, number of ventilation days, and physician and nurse staffing [10]. A level 1 ICU has a minimum of six beds and at least two intensivists; level 2 ICUs have a minimum of 12 beds and at least 0.35 full time equivalent (FTE) intensivists and 0.45 FTE house doctors per ICU bed; level 3 ICUs have a minimum of 12 beds and at least 0.45 FTE intensivists and 0.55 FTE house doctors per ICU bed [10, 16]. All types of hospitals and ICU levels were involved in this study.

### Individual and focus group interviews

Before the start of the study, we established contact with ICU physicians in six hospitals: two general, two teaching, and two academic hospitals. Through these six hospital contacts, ICU and ward physicians and nurses, and hospital managers were recruited by email for individual interviews. Before the study started there was no pre-existing relationship between the interviewers and the interviewees. We used a purposive sampling strategy to ensure a representative sample in terms of hospital type (general, teaching, academic, profession (ward nurse, ICU nurse, ward physician, ICU physician) and characteristics such as experience with ICU discharge process and type of general ward. A consultant of a medical insurance company was recruited from the Dutch umbrella organisation for medical insurance companies. The hospital managers and the employee of a medical insurance company were included to gain insight into possible financial or legal factors. In addition, two post-ICU patients were recruited through the hospital contacts. They were asked to participate by an ICU nurse and were interviewed in the presence of a relative during their stay in a general ward. We stopped including patients after interviewing two patients, because we were not able to gather any information about difficulties related to the ICU discharge process in both patient interviews. A topic guide was developed (Additional file 2) and pilot tested with one ICU physician. The individual interviews were conducted by a trained interviewer (NS), in the presence of one other researcher (AO), between April 2012 and December 2012. The number of interviews depended on the point of saturation, i.e., when no new information could be identified in the interviews [17].

To explore the barriers and facilitators identified in the individual interviews more in depth, we conducted four focus group interviews with: (1) ICU physicians, (2) ICU nurses, (3) general ward physicians, and (4) general ward nurses. Recruitment for the focus group interviews took place through snowball sampling: initially, the ICUs and general wards of the six initial hospitals were contacted and through these contacts, physicians and nurses of relevant wards in other hospitals were contacted and invited. The prospective professional participants were informed by email about the objective of the study, and were invited to participate.

The individual interviews with professionals took place at the participants’ place of work, the post-ICU patients were interviewed on the general ward, and the focus group interviews took place at a central location. The topic guide for the focus group interviews is included in Additional file 3. The focus group interviews were held in January 2013 and were led by a moderator (HW for focus groups 1 and 2, MZ for focus groups 3 and 4), respectively three (NS, MZ, AO) and two (NS, AO) other researchers were present, both to observe as well as to assist the moderator.

Audio of the individual and the focus group interviews was recorded and subsequently transcribed verbatim, and a note taker was present at the focus group interviews. The transcript of the focus group interviews was sent to the participants for corrections and additional comments.

### Questionnaire

The questionnaire contained 27 statements concerning barriers and facilitators for the implementation of improvement practices derived from the findings of the interviews. All statements used in the questionnaire were scored on a 6-point Likert scale ranging from ‘1 = strongly disagree’ to ‘6 = strongly agree’. A ‘not applicable’ answering option was also provided. Additionally, the questionnaire contained nine demographic questions and one open-ended question to enable respondents to provide comments. The online questionnaire was designed using LimeSurvey software, and its face validity was tested through two ICU physicians and two independent researchers. This test consisted of completion of the questionnaire and subsequent discussion of the questions together with three of the researchers (AO, NS, MZ).

In March 2013, an introductory e-mail containing the link to the online questionnaire was sent to all ICU physician members of the Dutch Society for Intensive Care (nearly all Dutch ICU physicians are a member of this society, *n* = 761) working in 90 hospitals, explaining the aim of the study, ensuring the anonymous and confidential handling of data, and inviting them to participate. A reminder was sent 2 weeks later. Informed consent was implied by completing and sending in the questionnaire.

### Data analysis

The interview and focus group transcripts were coded using Atlas.ti 6.2. The analysis was conducted using a framework approach, in which the objectives of the study are already set in advance and are shaped by the information requirements [17]. The framework used was described previously [18], and is based on three models related to implementing change [14, 19–21]. The barriers and facilitators found in the interviews were classified into the seven categories of the framework: intervention-related factors (e.g., feasibility), implementation-related factors (e.g., accessibility and support), patient-related factors (e.g., cognition), professional-related factors (e.g., behaviour and attitude), social factors (e.g., leadership and culture), organisation-related factors (e.g., financial resources), and society-related factors (e.g., regulations and laws). To further structure the results of the analysis, the barriers and facilitators were classified into subcategories.

The first five individual interviews were coded by NS, AO and MZ, after which any discrepancies were discussed until consensus was reached. A double analysis (NS and MZ) and subsequent discussion was also performed for the first focus group interview transcript. All other transcripts were coded by one researcher (NS).

The questionnaire results were analysed using SPSS 20. We recoded ‘strongly disagree’, ‘disagree’ and ‘somewhat disagree’ into ‘disagree’ (0). We recoded ‘strongly agree’, ‘agree’ and ‘somewhat agree’ into ‘agree’ (1). We ordered the statements in a table, in which 100% was the highest and 0% was the lowest measure of agreement. Subgroup analyses were carried out to study if there were differences in answers between subgroups based on demographic variables, i.e., gender, age, work experience, hospital type, ICU level (level 1 is the least advanced ICU and level 3 is the most advanced ICU), and number of ICU beds, using Pearson chi-square tests and logistic regression. A *p*-value < 0.05 was considered significant.

## Results

### Characteristics of the respondents

We conducted 23 semi-structured individual interviews (for participant characteristics, see Table 1). The interviews took between 11 and 74 min; the two patient interviews were relatively short (11 and 13 min). All invited persons agreed to participate, except for one ICU physician who declined for scheduling reasons.

**Table 1:** Characteristics of respondents

	Individual interviews	Focus group interviews
	(*n* = 23)	(*n* = 25)
Job Title		
ICU physician (%) ICU nurse (%) Ward physician (%) Ward nurse (%) Policy maker ^a^ (%) Patient (%)	5 (22) 5 (22) 3 (13) 5 (22) 3 (13) 2 (9)	5 (20) 7 (28) 5 (20) 8 (32) 0 (0) 0 (0)
Male (%)	10 (43)	8 (32)
Hospital type		
General (%) Teaching (%) Academic (%) Not applicable ^b^ (%)	6 (26) 4 (17) 10 (43) 3 (13)	5 (20) 10 (40) 10 (40) 0 (0)
Years clinical Experience in current specialty		
<5 years (%) 5–10 years (%) >10 years (%) Not applicable ^c^ (%)	8 (35) 5 (22) 5 (22) 5 (22)	5 (20) 7 (28) 13 (52) 0 (0)

^a^Policy makers: two hospital managers and one consultant of a medical insurance company

^b^One policy maker and the two patients were not affiliated to a hospital

^c^The policy makers and patients were not categorised

We conducted four focus group interviews (see Table 1). The focus group interviews took between 60 and 90 min. Seventeen ICU physicians were invited, five of whom participated in the focus group interview. Thirty-six general ward physicians were invited, five of whom participated in the interview. Twenty-five ICU nurses were invited, seven of whom took part in the interview. Twenty-five general ward nurses were invited, eight of whom participated in the interview. Most invited participants who declined, declined for scheduling reasons.

Of the 761 ICU physician members of the Dutch Society for Intensive Care, 166 physicians (21.8%) working in 64 different Dutch hospitals (71.1% of the total number of Dutch hospitals) completed the questionnaire. Respondent characteristics can be found in Table 2.

**Table 2:** Questionnaire respondent characteristics

	Respondents (*n* = 166)
Gender	
Male (%) Female (%) Missing (%)	106 (63.9) 57 (34.3) 3 (1.8)
Median Age (min-max)^a^	43 (31–64)
Median years of experience (min-max)^b^	7 (0–34)
Patient category	
Adults (%) Adults and children (%)	160 (96.4) 6 (3.6)
Hospital type	
General (%) Teaching (%) Academic (%) Missing (%)	50 (30.1) 70 (42.2) 45 (27.1) 1 (0.6)
ICU physician training hospital?	
Yes (%) No (%) Missing (%)	49 (29.5) 112 (67.5) 5 (3.0)
Median number of ICU beds (min-max)^a^	16 (6–58)

^a^1 missing

^b^8 missing

### Perceived barriers and facilitators

The participants in the individual and focus group interviews mentioned 66 barriers and facilitators for the implementation of improvement practices of the ICU discharge process (Table 3). Most factors were related to the intervention (*n* = 13), professional (*n* = 12) and organisation (*n* = 12). Only one patient-related factor and five factors related to the implementation process were mentioned.

**Table 3:** Perceived barriers and facilitators by the interview respondents

Category	Subcategory	Factor	B	F
Intervention	Credibility	Lack of evidence [0,4,6]	✓	
	Utility	Lack of details in intervention description [B:1,F:1]	✓	✓
	Advantage	Negative (B)/ positive (F) results experienced [B:6,F3]	✓	✓
		(Not) used when (not) useful [B:4,F:3]	✓	✓
		(Not) used when there is (no) need [B:6,8,F:4,5,6,7,8]	✓	✓
	Observability	(No) positive results shown [B:8,F:7]	✓	✓
	Feasibility	Does not work in practice [3,6,7]	✓	
		Not always possible to execute [3,4]	✓	
		Failed pilot test [8]	✓	
		Form not user friendly [4]	✓	
		Uniform policy is impossible [4]	✓	
		Policy tailored to each general ward is not feasible [4]	✓	
		Too many patients [7]	✓	
Implementation process	Accessibility	Intervention not converted into protocol [1]	✓	
		Protocol/policy available on intranet [1,2]		✓
	Clarity	Indistinct agreements surrounding intervention [4]	✓	
	Support	Initiative from care professionals [4]		✓
		Creating support among healthcare professionals		✓
Professional	Attitude	Opinion that intervention is no solution for structural problems [8]	✓	
		Opinion that formulating discharge criteria is (im)possible [B:1,F:1]	✓	✓
		Opinion that intervention is (not) useful [B:3,6,7,F:3,4]	✓	✓
		Negative attitude towards protocols or checklists [1,4]	✓	
		Negative attitude towards new or more forms [0,4]	✓	
		Negative attitude towards registration [0]	✓	
		Opinion that ICU physician is involved until hospital discharge [4]		✓
	Knowledge	Guideline or intervention is unknown [1,7]	✓	
		Physician has little knowledge about nursing discharge practices [3]	✓	
	Awareness	Awareness of possible unsafe practices [0,5]		✓
	Behaviour	Change of routines necessary [0,4]	✓	
	Skills	Lack of ICT skills [0,4]	✓	
Patient	Cognition	Communication impossible [5]	✓	
Social	Leadership	Care professionals are not involved in decision making [0]	✓	
		Prioritization of problem/implementation of intervention [0,8]	✓	
		Choices made in past [8]	✓	
	Culture	(No) culture of feedback [0,4]	✓	✓
		‘Ivory tower’-image of ICU [0]	✓	
		Cultural differences between wards [4]	✓	
	Collaboration	No multidisciplinary care [0]	✓	
		No or too little structural consultation with ward [4]	✓	
		Preconceived opinions against ICU professionals [0]	✓	
		ICU nurse performs tasks in general wards [0]	✓	✓
Organisational	Resources	Lack of man-hours/time [0,4,6,8]	✓	
		Ward physician is unavailable [4]	✓	
		Ward equipment is not yet set up [4]	✓	
		Lack of financial resources [8]	✓	
	Structure	Large (B) or small (F) hospital [B:0,7,F:7]	✓	✓
		ICU is ‘separated’ from hospital by architectural barriers [0]	✓	
		High turnover of physicians [3]	✓	
	ICT infrastructure	(No) hospital wide electronic patient file [B:4,F:4,5]	✓	✓
		No check, no summary as a result of one electronic patient file [4]	✓	
		Electronic patient file unclear/not user-friendly [5]	✓	
		Intervention is connected to electronic patient file [5]	✓	✓
	Policy	Confusion about which physician is responsible for patient [4]	✓	
Society	Financial support	No compensation by insurance company [0,6,8]	✓	
		Cuts are made to minimise expenditures [8]	✓	
		Confusion about financing structures [0,8]	✓	
	Financial incentives	Production is central [0]	✓	
	Regulations	Production instead of quality is performance measure [0]	✓	
		Variation in quality of step down beds due to a lack of policy [8]	✓	
	Other hospitals	Competition [7]	✓	✓
	Professional associations	Discussion whether ICU tasks can and should be performed in general wards by ICU professionals [0]	✓	
		Discussion about the reallocation of ICU tasks to general ward professionals [6]	✓	

[…] = interventions to which the factor is applicable; 0 = General; 1 = Dutch Intensive Care Society (NVIC) guideline; 2 = ICU discharge policies; 3 = Early discharge planning; 4 = Communication at handover; 5 = Medication reconciliation; 6 = Consulting ICU nurse; 7 = Monitoring of post-ICU patients; 8 = Step down beds

*Abbreviations*: *B* Barrier, *F* Facilitator

#### Intervention-related factors

Barriers mentioned by the interviewees related to the practices themselves were: lack of evidence, lack of details in the practice description, and lack of practical feasibility and applicability.

*“In some cases, the patient is ready for discharge early in the morning. If there is room in the receiving ward, the patient will leave a few hours later. Planning the discharge 24 h in advance is not necessary in these cases.” (ICU physician – individual interview)*

In the questionnaire, 65.7% of the questionnaire respondents considered planning an ICU discharge at least 24 h in advance not feasible. The respondents (77.1%) also thought that practice variation existed due to the lack of specific ICU discharge criteria, 69.3% of the questionnaire respondents would have liked more specific ICU discharge criteria, and 18.1% of the respondents thought it was impossible to set more specific discharge criteria.

*“That depends of course on when an ICU physician thinks a patient is not yet recovered enough to go to the general ward. There are no real criteria for that, for when a patient is ready for discharge. So it depends on what an ICU physician thinks whether or not a patient is discharged at that moment.” (ICU nurse – individual interview)*

In the interviews, lack of evidence was mentioned. The questionnaire results, however, showed that 74.1% of the respondents thought that little evidence was no barrier to implement an intervention.

#### Professional-related factors

In the questionnaire, 87.3% of the respondents thought that there was room to improve the communication between the ICU and the general ward. Professional-related factors mentioned in the interviews were negative attitudes towards checklists, towards more forms and towards registration in general.

*“These are things that you have memorised, because you have to work with them every day. You don’t need a list for that.” (ICU physician – individual interviews)*

Most questionnaire respondents (92.2%), however, considered a checklist useful at handover. A facilitator mentioned in the interviews was the involvement of an ICU physician with the patient until hospital discharge; 25.9% of the questionnaire respondents agreed that an intensivist should be involved with an ICU patient until hospital discharge.

#### Social factors

Social barriers mentioned by the interviewees were: lack of prioritisation by the management, no culture of feedback, no or little structural consultation with the general ward, and the ICU’s ‘island’ or ‘ivory tower’ image.

*“The ICU still remains a little bit of an island within the hospital. Whenever I have to call the ICU, I think: ‘I hope I have my story straight..’.” (Ward nurse – individual interview)*

In the questionnaire, 72.9% of the respondents thought that improving the ICU discharge process deserved more attention from the management, 41.0% found that ward professionals did not give feedback when the handover to the general ward was suboptimal, and 74.7% thought that they sometimes overestimated the capabilities of a general ward.

#### Patient-related factor

The only patient-related factor mentioned by the interviewees was that it is often impossible to communicate with ICU patients.

#### Organisation-related factors

Organisational barriers mentioned by the interviewees were: large hospital size, no electronic patient file, lack of financial resources, unavailability of the ward physician for face-to-face handover and lack of man hours/time.

*“It is bothersome, I think, to figure out who is the physician on the ward. I think that a face-to-face handover would be an improvement, but it costs a lot of time to call six physicians before you’ve got the right one.” (ICU physician – individual interview)*

In the questionnaire, 78.3% deemed an electronic patient file to be indispensable when making an up-to-date medication overview at ICU discharge, 65.1% considered the unavailability of the ward physician a barrier to performing a verbal handover, 49.4% found a lack of financial resources a barrier for implementing improvement interventions, 49.4% thought that it was organisationally impossible to create step down facilities, 45.8% considered monitoring post-ICU patients in general wards infeasible due to a limited number of available nurses, 25.3% regarded the size of their hospital as a barrier to improve the ICU discharge process, and 24.7% considered the amount of available nurses not sufficient for introducing a consulting ICU nurse position.

#### Society-related factors

One of the society-related barriers mentioned by the interviewees was the financial support by health insurance companies.

*“Health insurers should be realistic and make it possible to claim the costs of medium care facilities. At the moment we have no income from the medium care, and that is ridicules.” (ICU manager – individual interview)*

In the questionnaire, 49.4% of the respondents thought that a lack of financial resources was a barrier to implementing improvement interventions.

#### Implementation-related factors

Facilitators mentioned by the interviewees related to the implementation process was availability of protocols (such as handover checklists or discharge criteria) on the intranet and the support among professionals for implementing an ICU discharge practice.

*“The general ward worries whether the patient eats enough, whether he tries to stand and walk. We incorporate this in our handover, because they ask about it. But these points are not part of the standard discharge list. This could possibly be improved.” (ICU nurse – individual interview)*

In the questionnaire, 23.5% of the respondents stated that they did not have ICU discharge criteria in their ICU.

### Ranking

Table 4 shows the results of the questionnaire ordered from 100 to 0%. Three statements regarding communication received high rates: ‘I think that having a checklist to structure the verbal handover is useful’ (92.2% agreed), ‘I think that there is room to improve the communication between ICU and general ward’ (87.3% agreed), ‘I think that performing structured handover takes a lot of time’ (78.3% disagreed), and ‘I do sometimes overestimate the capabilities of a general ward’ (74.7% agreed). Three statements concerning discharge criteria received high rates: ‘I think that there are differences among ICU physicians in when they consider a patient ready for ICU discharge, because there are no specific ICU discharge criteria’ (77.1% agreed), ‘I think it is desirable to set more specific ICU discharge criteria’ (69.3% agreed), and ‘I think it is possible to set more specific ICU discharge criteria’ (74.7% disagreed). Furthermore, 41.0% of the questionnaire respondents disagreed with the statement ‘In my experience ward professionals give feedback when the handover to the general ward was suboptimal’ and 72.9% agreed with the statement ‘Improving the ICU discharge deserves more attention from the management’.

**Table 4:** Results of statements used in the questionnaire (*n* = 166)

Category	Subcategory	Statement	Agree (%)	Disagree (%)	NA^a^ (%)
P	Attitude	I think that having a checklist to structure the verbal handover is useful.^c^	153 (92.2)	7 (4.2)	6 (3.6)
P	Attitude	I think that there is room to improve the communication between ICU and general ward.^c, g^	145 (87.3)	19 (11.4)	2 (1.2)
I	Resources	I experience enough demand from the ward to implement/sustain the consulting ICU nurse position.	138 (83.1)	20 (12.0)	8 (4.8)
O	ICT infrastructure	I think that when making an up-to-date medication overview at ICU discharge a electronic patient file is indispensable. ^d^	130 (78.3)	32 (19.3)	4 (2.4)
I	Utility	I think that there are differences between intensivists in when they deem a patient ready for ICU discharge, because there are no specific ICU discharge criteria.	128 (77.1)	32 (19.3)	6 (3.6)
S	Collaboration	I do sometimes overestimate the possibilities in a general ward.^e^	124 (74.7)	38 (22.9)	4 (2.4)
S	Leadership	I think that improving the ICU discharge process deserves more attention from the management.^e, f^	121 (72.9)	40 (24.1)	5 (3.0)
O	Resources	I think that implementing improvement interventions takes a lot of energy and time.	117 (70.5)	46 (27.7)	3 (1.8)
I	Utility	I think it is desirable to set more specific ICU discharge criteria.	115 (69.3)	48 (28.9)	3 (1.8)
I	Feasibility	I think that planning the discharge of an ICU patient 24 h in advance is not feasible in daily practice, because the time between the decision to discharge and actual handover is often less than 24 h.^d^	109 (65.7)	54 (32.5)	3 (1.8)
O	Resources	A major reason for not performing a verbal handover between physicians is the fact that the ward physician is often not available.	108 (65.1)	50 (30.1)	8 (4.8)
S	Culture	In my experience ward professional do give feedback when the handover to the general ward was suboptimal,	92 (55.4)	68 (41.0)	6 (3.6)
O	Resources	I think that a lack of financial resources is a barrier for implementing improvement interventions.	82 (49.4)	79 (47.6)	5 (3.0)
O	Resources	In my opinion it is organisationally impossible to make step down facilities.^d^	82 (49.4)	70 (42.2)	14 (8.4)
O	Resources	I think that because of an insufficient nursing staff it is not feasible to monitor post-ICU patient on the wards. ^b^	76 (45.8)	83 (50.0)	7 (4.2)
Sy	Professional associations	I think that relocating ICU tasks to the wards by a consulting ICU nurse is not desirable. ^c^	65 (39.2)	100 (60.2)	1 (0.6)
I	Credibility	I think the ICU discharge criteria as described in the NVIC guideline are sufficiently based on scientific evidence.	62 (37.3)	79 (47.6)	25 (15.1)
I	Utility	I think that the ICU discharge criteria as described in the NVIC guideline are unclear.	58 (34.9)	91 (54.8)	17 (10.2)
P	Attitude	I think that intensivists should be involved in care for ICU patients until they are discharged from the hospital.	43 (25.9)	123 (74.1)	0 (0.0)
I	Credibility	If there is no scientific evidence for an intervention, I think that this intervention should not be implemented into daily practice.	42 (25.3)	123 (74.1)	1 (0.6)
O	Structure	I think that the size of my hospital makes it more difficult to improve the ICU discharge process.^c, e, f, g^	42 (25.3)	115 (69.3)	9 (5.4)
O	Resources	I think the current nursing staff is not sufficient for introducing a consulting ICU nurse position.	41 (24.7)	117 (70.5)	8 (4.8)
IP	Accessibility	I’ve never seen written ICU discharge criteria in our ICU.^c, d^	39 (23.5)	124 (74.7)	3 (1.8)
I	Feasibility	I think that performing structured handover takes a lot of time.	34 (20.5)	130 (78.3)	2 (1.2)
I	Credibility	Because little is known about causes of ICU readmissions, we can’t do anything about this problem.	31 (18.7)	134 (80.7)	1 (0.6)
I	Utility	I think it is impossible to set more specific ICU discharge criteria.	30 (18.1)	124 (74.7)	12 (7.2)
P	Attitude	I think that the sickest patient should be the priority of the intensivist. Patients who are almost ready for ICU discharge are of less importance.^f^	21 (12.7)	143 (86.1)	2 (1.2)

*Abbreviations*: *NA* not applicable, *P* professional, *I* intervention, *O* organisational, *S* social, *Sy* society, *IP* implementation process

^a^missing data was also grouped in this category

^b^Answers influenced by gender

^c^Answers influenced by age

^d^Answers influenced by work experience

^e^Answers influenced by hospital type

^f^Answers influenced by ICU level

^g^Answers influenced by number of ICU beds

### Subgroup analyses

We found significant differences in answers to five statements among respondents in different age categories. For example, significantly more respondents in the category ≤40 years thought that there was room to improve the communication between ICU and general ward than respondents in the category 41–50 years (96.6% vs. 82.6%, *p* = 0.023). We found significant differences in answers to four statements among respondents in different categories of number of years experience. For example, significantly more respondents with work experience of ≤5 years thought that it was impossible to organise step down facilities than respondents with a work experience of more than 15 years (67.3% vs. 33.3%, *p* = 0.009). Respondents from academic hospitals and level 2 and 3 ICUs significantly more often concluded that the ICU discharge process deserved more attention from the management than respondents from general hospitals or level 1 and level 2 ICUs (hospital type: academic 86.0% vs. general 61.2%, *p* = 0.010; ICU level: level 2 83.3% and level 3 78.2% vs. level 1 57.1%, *p* =0.010 and *p* = 0.024). Respondents from general hospitals had the opinion that they overestimated the possibilities on a ward significantly less often than respondents from academic and teaching hospitals (63.3% vs. an average of 77.0%, *p* = 0.023). The cross tables of the subgroup analyses can be found in Additional file 4.

## Discussion

### Main findings and related literature

In this study, 66 barriers and facilitators were found for the implementation of ICU discharge interventions, which were directed towards the intervention itself; the opinion, skills and knowledge of the professional executing the intervention; social factors, such as culture, communication, collaboration and leadership; and factors concerning available resources, organisational structures and ICT infrastructures. Important barriers were related to communication between ICU and general ward professionals, lack of specific discharge criteria and organisational factors, such as lack of priority by the management and cultural factors. Only one patient-related factor was identified. This may have been caused by an inactive role of many patients during transition due to reduced consciousness and a fragile state of health. Implementation-related factors were also limited, because many practices evolved over time and did therefore not have an explicit implementation process.

Almost 90% of the questionnaire respondents concluded that the communication between ICU and general ward could be improved and deserved more attention from the management (72.9%). Patient discharge summaries are an important communication tool which can prioritize or highlight certain information [1]. Kripalani et al. stated that the traditional methods of completing and delivering discharge summaries are suboptimal for communicating timely, accurate, and medically important patient data between hospital-based and primary care physicians [22]. They suggested several steps to improve communication and the quality of discharge summaries by for example computer-generated summaries and standardized formats. Cheung et al. also saw a lack of standardisation as a barrier to optimal handoff between shifts [23]. Checklists are often used to structure and therefore improve the handover communication and over 90% of the questionnaire respondents considered a checklist useful. In the interviews, however, we identified a negative attitude towards checklists as a barrier to implementation and use. The questionnaire respondents’ positive attitude towards checklists is in contrast with an interview study of Russ and colleagues, in which resistance and noncompliance from particularly senior clinicians was the most common barrier to using a checklist [24].

The communication between particularly ward nurses and ICU may also have been hindered by the ‘ivory tower’ or ‘island’ image of the ICU that the interviewees mentioned. This image is caused by cultural differences between the ICU and the general wards and physical separation of the ICU from the rest of the hospital, and leads to unfamiliarity and misunderstandings. Riesenberg et al. also reported communication barriers related to social structures and hierarchies in a research on nursing handoffs [25]. Lin et al. stated that teamwork involves shared organisational goals and coordination among team members and across teams to improve ICU discharge [12]. In a later article by Lin et al., they reported that a lack of communication across departments and different teams’ competing priorities contributed to ICU discharge delays [26].

This social barrier is also related to the perceived lack of a culture of feedback (i.e., professionals not being held accountable for suboptimal communication) by the questionnaire respondents. The presence or absence of a culture of feedback affects the implementation and use of discharge practices and the quality of handover at ICU discharge in general. Hesselink and colleagues researched hospital discharge, and described that feedback is not always feasible due to, for instance, time constraints, but also because feedback is believed to be inappropriate [27].

According to almost 70% of our questionnaire respondents, more specific ICU discharge criteria are desirable. The current situation, no specific criteria, leads to differences in when ICU physicians deem a patient ready for discharge. A literature review showed that written ICU discharge guidelines are often missing in ICUs, and noted that the guidelines used are often based on consensus instead of empirical evidence [12]. Currently, discharge criteria are mostly geared towards determining when a patient is no longer in need of ICU care. There is, however, a significant gap between when a patient is no longer in need of ICU care and when a patient can be safely cared for in a general ward. ICUs and general wards differ significantly in terms of nurse-to-patient ratio (in other words, how often a nurse can check on the patient), as well as the knowledge and skills that ward nurses need to perform complex nursing interventions [28]. The discharge of a patient from the ICU to a general ward usually means a heavy workload for the ward nurses, and moral distress when they are not able to give each patient in the ward the care he or she needs [29]. The capability of general wards to care for complex patients is not always clear to ICU professionals; in our study, almost 75% of ICU physicians said they sometimes overestimate care possibilities on general wards. This may result in early discharges and adverse events after ICU discharge. The same was found in the discharge from hospital to community care setting: Hesselink and colleagues found that hospital staff was unacquainted with care in the community and did not adequately anticipate the needs of the community care providers [30].

### Strengths and limitations

Methodological strengths of this study are the use of a mixed methods approach and a theoretical framework to analyse the interviews [18]. Semi-structured face-to-face interviews were used to explore barriers and facilitators in-depth [17]. In the subsequent focus groups, the group dynamic and interaction among participants helped to further explore and clarify participants’ views on barriers and facilitators [17]. To verify the broad exploration of barriers and facilitators in the interviews in a larger group and to quantify the results, we used an online questionnaire [31]. By including different types of professionals and managers from different types of hospitals and different wards in our interviews, we ensured a breadth of perspectives, increasing the generalisability of our research. However, differences in cultures and health systems among countries may negatively impact the generalisability of the results.

Our study had several other limitations. We asked the interview participants about eight practices, which made it difficult to explore all practices in depth in each interview. Furthermore, although almost every professional invited for an individual interview agreed to participate, only 24% of the professionals invited to the focus groups agreed to participate. This was mainly caused by scheduling, since the focus group interviews were scheduled by the researchers and the individual interviews were planned at the convenience of the participant. Furthermore, the individual interviews took place at the workplace of the participant and the participants in the focus group interviews had to travel to the interview location.

The response rate to the questionnaire of 21.8% was quite modest. However, taking into account the proportion of hospitals with at least one respondent, we included nearly three-quarters of Dutch hospitals. We could not access demographic data of the non-respondents for reasons of confidentiality and were therefore unable to analyse the representativeness of our respondents. Moreover, no postal addresses or telephone numbers were available to us, so we could reach respondents only via email. Therefore, we were unable to increase the response rate by using additional methods to reach out to potential respondents. Although a low-response rate increases the potential for non-response bias, research by Kellerman et al. suggests that the risk of non-response bias may be lower in survey research among physicians than among other populations, possibly since physicians are a relatively homogenous group [32]. In previous studies analysing non-respondents of survey research, non-response bias was suggested in research in which women, recently licensed physicians and younger physicians were more likely to respond [33, 34]. Our study population, however, consisted of a varied sample in terms of age, experience and gender.

The interviews with the patients did not result in any findings, mostly because the patient and the relative present were not aware of the different ICU discharge practices. Therefore, we decided to stop including patients after two interviews. This may have influenced the generalisability of the results, because they are mainly based on health professionals’ opinions.

### Implications for practice

To decrease practice variation, it is necessary for ICUs to come to an agreement about discharge criteria and the ICU discharge process in general. Capturing these agreements in a guideline could be helpful. The results of this study provide input to improve the existing Dutch national guideline for ICU admission and discharge [35]. An important aspect to consider when evaluating whether or not a patient can be safely discharged is the current capacity of the general ward. Characteristics such as number and skill mix of ward staff, and care burden of other patients already on the general ward need to be taken into account. To avoid overestimation of the capabilities of a general ward by the ICU, agreements should be made between ICU and each general ward on ward-specific discharge criteria. These criteria should be evaluated regularly, as skill mix and resources in general wards may change, in order to ensure the safety of post-ICU patients in general wards.

The communication between ICU and general ward needs to be improved, and in this study, most ICU physicians considered a checklist a useful tool. Structurally evaluating necessary handover information and communication preferences is of the essence in organising a safe and efficient ICU discharge process [1, 22, 23].

The process of implementing practices could be enhanced by stimulating a culture of professional feedback, in order to create learning experiences from suboptimal handover situations [27, 36]. To decrease the ‘island’ image of the ICU, to reduce unfamiliarity and misunderstandings and to improve cooperation between ICU and general ward, team training, multidisciplinary meetings, cross-over internships and improvement of leadership could be used [12].

### Implications for research

To be able to set specific discharge criteria, more research is necessary to gain knowledge about the characteristics of readmitted patients, but also about organisational processes that may influence and predict readmission. This information is needed to develop a screening instrument to identify patients at risk for readmission or post-ICU mortality. Subsequently, interventions could be developed tailored to these specific groups of high-risk patients to avoid adverse events.

In our research we identified few patient factors influencing the ICU discharge process. The current rise in ICU aftercare, such as support from and visits to the ICU after a patient is discharged home, could be used to gain insight in patient experiences during the ICU discharge process. Their experiences are necessary to optimise the ICU discharge process and to provide continuity of care for these vulnerable patients.

## Conclusion

Based on the barriers and facilitators found in this study, improving the handover communication, formulating specific discharge criteria, stimulating a culture of feedback, and preventing overestimation of the general ward are important to effectively improve the ICU discharge process.

## Key messages

- To decrease practice variation, it is necessary for ICU and general ward to agree on discharge criteria and the ICU discharge process in general.
- An important aspect to consider when evaluating whether or not a patient can be safely discharged is the current capacity of the general ward; characteristics such as number and skill mix of ward staff, and care burden of other patients already on the general ward need to be taken into account.
- To effectively improve the ICU discharge process, improving handover communication, formulating specific discharge criteria, stimulating a culture of feedback, and preventing overestimation of the general ward are important.

## Additional files

Additional file 1: Consolidated criteria for reporting qualitative studies (COREQ). (PDF 609 kb)

Additional file 2: Example of the individual interview guide. (PDF 424 kb)

Additional file 3: Example of the focus group interview guide. (PDF 275 kb)

Additional file 4: Subgroup analyses. (PDF 468 kb)

## Abbreviations

COREQ: Consolidated criteria for reporting qualitative studies
ICT: Information and communications technology
ICU: Intensive care unit
