# Supplementary material for: Barriers and facilitators to improve safety and efficiency of the ICU discharge process: a mixed methods study
Source: BMC Health Serv Res. 2017 Apr 4;17:251. doi: 10.1186/s12913-017-2139-x (PMC5381117; doi:10.1186/s12913-017-2139-x)
Supplement: Supplementary file 1 — Consolidated criteria for reporting qualitative studies (COREQ). (PDF 609 kb) [file 12913_2017_2139_MOESM1_ESM.pdf]

Additional file 1. Consolidated criteria for reporting qualitative studies (COREQ)

Consolidated criteria for reporting qualitative studies (COREQ): 32-item checklist

Developed from:
Tong A, Sainsbury P, Craig J. Consolidated criteria for reporting qualitative research (COREQ): a 32-item checklist for interviews and focus groups. *International Journal for Quality in Health Care*. 2007. Volume 19, Number 6: pp. 349 – 357

**No. Item**	**Guide questions/description**	**Reported in section**
**Domain 1: Research team and reflexivity**		
Personal Characteristics		
1. Interviewer/facilitator	Which author/s conducted the interview or focus group?	5 (individual and focus group interviews)
2. Credentials	What were the researcher's credentials? E.g. PhD, MD	5 (individual and focus group interviews)
3. Occupation	What was their occupation at the time of the study?	Both interviewers are researchers
4. Gender	Was the researcher male or female?	Both interviewers are female
5. Experience and training	What experience or training did the researcher have?	5 (individual and focus group interviews)
Relationship with participants		
6. Relationship established	Was a relationship established prior to study commencement?	5 (individual and focus group interviews)
7. Participant knowledge of the interviewer	What did the participants know about the researcher? e.g. personal goals, reasons for doing the research	5 (individual and focus group interviews)
8. Interviewer characteristics	What characteristics were reported about the inter viewer/facilitator? e.g. Bias, assumptions, reasons and interests in the research topic	4 (individual and focus group interviews)
**Domain 2: study design**		
Theoretical framework		
9. Methodological orientation and Theory	What methodological orientation was stated to underpin the study? e.g. grounded theory, discourse analysis, ethnography, phenomenology, content analysis	6 (data analysis)
Participant selection		

10. Sampling	How were participants selected? e.g. purposive, convenience, consecutive, snowball	4-5 (individual and focus group interviews)
11. Method of approach	How were participants approached? e.g. face-to-face, telephone, mail, email	4-5 (individual and focus group interviews)
12. Sample size	How many participants were in the study?	8 (characteristics of respondents)
13. Non-participation	How many people refused to participate or dropped out? Reasons?	8 (characteristics of respondents)
Setting		
14. Setting of data collection	Where was the data collected? e.g. home, clinic, workplace	4-5 (individual and focus group interviews)
15. Presence of non-participants	Was anyone else present besides the participants and researchers?	5 (individual and focus group interviews)
16. Description of sample	What are the important characteristics of the sample? e.g. demographic data, date	8 (characteristics of respondents)
Data collection		
17. Interview guide	Were questions, prompts, guides provided by the authors? Was it pilot tested?	4-5 (individual and focus group interviews)
18. Repeat interviews	Were repeat inter views carried out? If yes, how many?	Not applicable
19. Audio/visual recording	Did the research use audio or visual recording to collect the data?	4-5 (individual and focus group interviews)
20. Field notes	Were field notes made during and/or after the interview or focus group?	4-5 (individual and focus group interviews)
21. Duration	What was the duration of the inter views or focus group?	8 (characteristics of respondents)
22. Data saturation	Was data saturation discussed?	5 (individual and focus group interviews)
23. Transcripts returned	Were transcripts returned to participants for comment and/or correction?	5 (individual and focus group interviews)
**Domain 3: analysis and findings**		
Data analysis		
24. Number of data coders	How many data coders coded the data?	7 (data analysis)
25. Description of the coding tree	Did authors provide a description of the coding tree?	26 (table 3)
26. Derivation of themes	Were themes identified in advance	6 (data analysis)

	or derived from the data?	
27. Software	What software, if applicable, was used to manage the data?	6 (data analysis)
28. Participant checking	Did participants provide feedback on the findings?	No
Reporting		
29. Quotations presented	Were participant quotations presented to illustrate the themes/findings? Was each quotation identified? e.g. participant number	9-12 (results)
30. Data and findings consistent	Was there consistency between the data presented and the findings?	9-12 (results)
31. Clarity of major themes	Were major themes clearly presented in the findings?	9-12 (results)
32. Clarity of minor themes	Is there a description of diverse cases or discussion of minor themes?	9-12 (results)
