# Supplementary material for: Barriers and facilitators to improve safety and efficiency of the ICU discharge process: a mixed methods study
Source: BMC Health Serv Res. 2017 Apr 4;17:251. doi: 10.1186/s12913-017-2139-x (PMC5381117; doi:10.1186/s12913-017-2139-x)
Supplement: Supplementary file 2 — Example of the individual interview guide. (PDF 424 kb) [file 12913_2017_2139_MOESM2_ESM.pdf]

Additional file 2 Example of the individual interview guide

Interview Guide ICU physicians

Introduction

- Short introduction to the study
- Ask permission to record and transcribe the interview
- Introduction of researchers and interviewee (name, function title, years of experience)

ICU discharge process

- How is the decision to discharge a patient made?
    - Who is involved?
    - Is the receiving ward consulted?
- Are there set discharge criteria?
    - If yes, what are they?
    - If yes, are they used in practice?
    - If no, on what factors is a decision to discharge a patient based?
- Are there non-medical reasons to discharge or to delay a discharge?
- Do financial aspects and budget cuts impact discharge decisions?
    - Are there any financial incentives to discharge a patient early or delay discharge?
- What are the agreements with receiving wards?
    - How and when is the receiving ward informed about the discharge of an ICU-patient?
    - Are these agreements set?
- How does the handover of information between the ICU and receiving ward go?
    - How does the communication go?
    - In what way could the handover of information and communication be improved?

Policy

- Is policy concerning ICU discharge written down?
    - If no, why not?
- Is the Dutch guideline concerning ICU admission and discharge translated into policy and/or a protocol?
    - If yes, is it used in practice?
    - If no, why not?
- Is the policy translated into a checklist?
    - If yes, is it used in every discharge?
    - If no, why not?

ICU discharge practices

- Are step down facilities available in your hospital?
    - If no, why not?
- Does an ICU physician or nurse keep track of the number of available ICU beds?
    - If no, why not?
    - If yes, does everyone use this person?

    - How is this implemented? Are there agreements made with general wards?
- Is a recent medication overview made at discharge, which includes allergy information and stopped and changed home medications?
    - If no, why not?
    - If yes, is this verified with the patient or his/her relatives?
- Are post-ICU patients structurally visited on the ward by an ICU physician or nurse?
    - If no, why not?
    - If yes, are all patients visited or only a specific group of patients?
- Is advice or help from ICU nurses with certain activities available to the receiving ward ?
    - If no, why not?
    - If yes, do all receiving wards use it?
- Does your hospital have a medical emergency team or outreach team?
    - If no, who not?
    - If yes, do all receiving wards use it?
    - Are patients discharge earlier due to this intervention?

Concluding

- Which factors contribute to a ICU readmission?
- What could be improved concerning the discharge of ICU patients?
- Do you have suggestions how it could be improved?
- What are barriers and facilitators for implementing improvements?
