# Supplementary material for: Barriers and facilitators to improve safety and efficiency of the ICU discharge process: a mixed methods study
Source: BMC Health Serv Res. 2017 Apr 4;17:251. doi: 10.1186/s12913-017-2139-x (PMC5381117; doi:10.1186/s12913-017-2139-x)
Supplement: Supplementary file 3 — Example of the focus group interview guide. (PDF 275 kb) [file 12913_2017_2139_MOESM3_ESM.pdf]

Additional file 3 Example of the focus group interview guide

1. What are the causes of (variation between hospitals in) mortality and readmissions after ICU discharge?
2. What are solutions to prevent mortality and readmissions after ICU discharge?
3. Which practices are most effective according to you?
4. Why are these practices not yet implemented in every hospital? What are barriers and facilitators for implementation and use?
