# Supplementary material for: Barriers and facilitators to improve safety and efficiency of the ICU discharge process: a mixed methods study
Source: BMC Health Serv Res. 2017 Apr 4;17:251. doi: 10.1186/s12913-017-2139-x (PMC5381117; doi:10.1186/s12913-017-2139-x)
Supplement: Supplementary file 4 — Subgroup analyses. (PDF 468 kb) [file 12913_2017_2139_MOESM4_ESM.pdf]

Additional file 4 Subgroup analyses

Question 3: 'I think that improving the ICU discharge process deserves more attention from the management.'				
**Hospital type**	Disagree	Agree	Total	logistic regression (p)
Academic	6 (14.0)	37 (86.0)	43 (100)	0.010
Teaching	15 (22.1)	53 (77.3)	68 (100)	0.052
General	19 (38.8)	30 (61.2)	49 (100)	
Total	40 (25.0) (ref)	120 (75.0)	160 (100)	
Pearson Chi-square (p)	.018			

Question 3: 'I think that improving the ICU discharge process deserves more attention from the management.'				
**ICU level**	Disagree	Agree	Total	logistic regression (p)
Level 1	15 (42.9) (ref)	20 (57.1)	35 (100)	
Level 2	8 (16.7)	40 (83.3)	48 (100)	0.010
Level 3	17 (21.8)	61 (78.2)	78 (100)	0.024
Total	40 (24.8)	121 (75.2)	161 (100)	
Pearson Chi-square (p)	.017			

Question 4: 'I think that the size of my hospital makes it more difficult to improve the ICU discharge process.'				
**Age**	Disagree	Agree	Total	logistic regression (p)
=<40 (%)	34 (60.7) (ref)	22 (39.3)	56 (100)	
41-50 (%)	54 (80.6)	13 (19.4)	67 (100)	0.017
>50 (%)	26 (78.8)	7 (21.2)	33 (100)	0.083
Total	114 (73.8	42 (26.9)	156 (100)	
Pearson Chi-square (p)	.033			

Question 4: 'I think that the size of my hospital makes it more difficult to improve the ICU discharge process.'				
**Hospital type**	Disagree	Agree	Total	logistic regression (p)
Academic	19 (42.2) (ref)	26 (57.8)	45 (100)	
Teaching	56 (80.0)	14 (20.0)	70 (100)	0.000
General	39 (95.1)	2 (4.9)	41 (100)	0.000
Total	114 (73.1)	42 (26.9)	156 (100)	
Pearson Chi-square (p)	.000			

Question 4: ‘I think that the size of my hospital makes it more difficult to improve the ICU discharge process.’				
**ICU level**	Disagree	Agree	Total	logistic regression (p)
Level 1	23 (82.1)	5 (17.9)	28 (100)	0.029
Level 2	44 (93.6)	3 (6.4)	48 (100)	0.000
Level 3	48 (58.5) (ref)	34 (41.5)	82 (100)	
Total	115 (73.2)	42 (26.8)	157 (100)	
Pearson Chi-square (p)	.000			

Question 4: ‘I think that the size of my hospital makes it more difficult to improve the ICU discharge process.’				
**No. ICU beds**	Disagree	Agree	Total	logistic regression (p)
=< 10	32 (94.1)	2 (5.9)	34 (100)	0.001
11-25	55 (78.6)	15 (21.4)	70 (100)	0.003
>25	28 (52.8) (ref)	25 (47.2)	53 (100)	
Total	115 (73.2)	42 (26.8)	157 (100)	
Pearson Chi-square (p)	.000			

Question 5: ‘I think that the sickest patient should be the priority of the ICU physician. Patients who are almost ready for ICU discharge are of less importance.’				
**ICU level**	Disagree	Agree	Total	logistic regression (p)
Level 1	28 (80.0)	7 (20.0)	35 (100)	0.026
Level 2	46 (97.9) (ref)	1 (2.1)	47 (100)	
Level 3	69 (84.1)	13 (15.9)	82 (100)	0.41
Total	143 (87.2)	21 (12.8)	164 (100)	
Pearson Chi-square (p)	.029			

Question 8: ‘I think that there is room to improve the communication between ICU and general ward.’				
**Age**	Disagree	Agree	Total	logistic regression (p)
Age =<40 (%)	2 (3.4) (ref)	57 (96.6)	59 (100)	
Age 41-50 (%)	12 (17.4)	57 (82.6)	69 (100)	0.023
Age >50 (%)	5 (14.3)	30 (85.7)	35 (100)	0.072
Total	19 (11.7)	144 (88.3)	163 (100)	
Pearson Chi-square (p)	.042			

Question 8: 'I think that there is room to improve the communication between ICU and general ward.'				
**No. ICU beds**	Disagree	Agree	Total	logistic regression (p)
=< 10	9 (22.5) (ref)	31 (77.5)	40 (100)	
11-25	5 (7.1)	65 (92.9)	70 (100)	0.027
>25	5 (9.4)	48 (90.6)	53 (100)	0.089
Total	19 (11.7)	144 (88.3)	163 (100)	
Pearson Chi-square (p)	.045			

Question 9: 'I do sometimes overestimate the possibilities on a general ward.'				
**Hospital type**	Disagree	Agree	Total	logistic regression (p)
Academic	8 (18.2)	36 (81.8)	44 (100)	0.050
Teaching	11 (16.2)	57 (83.8)	68 (100)	0.013
General	18 (36.7) (ref)	31 (63.3)	49 (100)	
Total	37 (23.0)	124 (77.0)	161 (100)	
Pearson Chi-square (p)	.023			

Question 11: 'I've never seen written ICU discharge criteria on our ICU.'				
**Age**	Disagree	Agree	Total	logistic regression (p)
=<40 (%)	35 (58.3) (ref)	25 (41.7)	60 (100)	
41-50 (%)	60 (88.2)	8 (11.8)	68 (100)	0.000
>50 (%)	28 (82.4)	6 (17.6)	34 (100)	0.021
Total	123 (75.9)	39 (24.1)	162 (100)	
Pearson Chi-square (p)	.000			

Question 11: 'I've never seen written ICU discharge criteria on our ICU.'				
**Work experience**	Disagree	Agree	Total	logistic regression (p)
=<5 (%)	34 (56.7)	26 (43.3)	60 (100)	0.000
6-15 (%)	66 (91.7) (ref)	6 (8.3)	72 (100)	
>15 (%)	17 (73.9)	6 (26.1)	23 (100)	0.034
Total	117 (75.5)	38 (24.5)	155 (100)	
Pearson Chi-square (p)	.000			

Question 16: 'I think that planning the discharge of an ICU patient 24 hours in advance is not feasible in daily practice, because the time between the decision to discharge and actual handover is often less than 24 hours.'				
**Work experience**	Disagree	Agree	Total	logistic regression (p)
=<5 (%)	15 (25.4)	44 (74.6)	59 (100)	0.022
6-15 (%)	32 (45.1) (ref)	39 (54.9)	71 (100)	
>15 (%)	4 (16.0)	21 (84.0)	25 (100)	0.014
Total	51 (32.9)	104 (67.1)	155 (100)	
Pearson Chi-square (p)	.009			

Question 17: 'I think that when making an up-to-date medication overview at ICU discharge a electronic patient file is indispensable.'				
**Work experience**	Disagree	Agree	Total	logistic regression (p)
=<5 (%)	7 (11.9)	52 (88.1)	59 (100)	0.009
6-15 (%)	23 (31.9) (ref)	49 (68.1)	72 (100)	
>15 (%)	2 (8.7)	21 (91.3)	23 (100)	0.041
Total	32 (20.8)	122 (79.2)	154 (100)	
Pearson Chi-square (p)	.006			

Question 18: 'I think a checklist to structure the verbal handover is useful.'				
**Age**	Disagree	Agree	Total	logistic regression (p)
=<40 (%)	0 (0.0)	58 (100)	58 (100)	-*
41-50 (%)	7 (10.4) (ref)	60 (89.6)	57 (100)	
>50 (%)	0 (0.0)	34 (100)	34 (100)	
Total	7 (4.4)	152 (95.6)	159 (100)	-*
Pearson Chi-square (p)	.007			

* Impossible to perform logistic regression because of empty fields.

Question 22: 'In my opinion it is organisationally impossible to organise step down facilities.'				
**Work experience**	Disagree	Agree	Total	logistic regression (p)
=<5 (%)	18 (32.7)	37 (67.3)	55 (100)	0.009
6-15 (%)	34 (49.3)	35 (50.7)	69 (100)	0.166
>15 (%)	14 (66.7) (ref)	7 (33.3)	21 (100)	
Total	66 (45.5)	79 (54.5)	145 (100)	
Pearson Chi-square (p)	.020			

Question 25: 'I think that relocating ICU tasks to the wards by a consulting ICU nurse is not desirable.'				
**Age**	Disagree	Agree	Total	logistic regression (p)
=<40 (%)	44 (73.3) (ref)	16 (26.7)	60 (100)	
41-50 (%)	40 (58.0)	29 (42.0)	69 (100)	0.070
>50 (%)	16 (45.7)	19 (54.3)	35 (100)	0.008
Total	100 (61.0)	64 (39.0)	164 (100)	
Pearson Chi-square (p)	.023			

Question 26: 'I think that because of an insufficient nursing staff it is not feasible to monitor post-ICU patient on the wards.'			
**Gender**	Disagree	Agree	Total
Male (%)	58 (58.6)	41 (41.4)	99 (100)
Female (%)	24 (42.1)	33 (57.9)	56 (100)
Total	82 (52.6)	74 (47.7)	156 (100)
Pearson Chi-square (p)	.047		
